# Survival of pneumococcus on hands and fomites

**DOI:** 10.1186/1756-0500-1-112

**Published:** 2008-11-13

**Authors:** Heidi Smith-Vaughan, Faith Crichton, Jemima Beissbarth, Peter S Morris, Amanda J Leach

**Affiliations:** 1 Child Health Division, Menzies School of Health Research, Darwin, Australia; 2 Institute of Advanced Studies, Charles Darwin University, Darwin, Australia; 3 NT Clinical School, Flinders University, Darwin, Australia

## Abstract

**Background:**

Pneumococcal hand contamination in Indigenous children in remote communities is common (37%). It is not clear whether this requires frequent inoculation, or if pneumococci will survive on hands for long periods of time. Thus the aim of this study was to determine the survival time of pneumococci on hands and fomites.

**Findings:**

The hands of 3 adult volunteers, a glass plate and plastic ball were inoculated with pneumococci suspended in two different media. Survival at specified time intervals was determined by swabbing and re-culture onto horse blood agar. Pneumococci inoculated onto hands of volunteers were recovered after 3 minutes at 4% to 79% of the initial inoculum. Recovery from one individual was consistently higher. By one hour, only a small number of pneumococci were recovered and this was dependent on the suspension medium used. At subsequent intervals and up to 3 hours after inoculation, < 10 colony forming units were recovered from hands. On a glass plate, pneumococcal numbers dropped an average 70% in the two hours after inoculation. Subsequently, < 100 colony forming units were recovered up to 15 hours after inoculation.

**Conclusion:**

The poor survival of pneumococci on hands suggests that the high prevalence of pneumococcal hand contamination in some populations is related to frequent inoculation rather than long survival. It is plausible that hand contamination plays a (brief) role in transmission directly, and indirectly through contamination via fomites. Regular hand washing and timely cleansing or removal of contaminated fomites may aid control of pneumococcal transmission via these routes.

## Background

Indigenous Australian children suffer exceedingly high rates of otitis media (OM). Recent surveys report average tympanic membrane perforation rates of 24% for Indigenous children living in remote communities [1] – far exceeding the rate of 4% described by the World Health Organisation as a public health emergency [2]. OM in Indigenous children has an early age of onset, often occurring within weeks of birth as a result of early and repeated acquisition of multiple respiratory pathogens [3].

*Streptococcus pneumoniae *(pneumococcus) commonly colonises the nasopharynx of children. Cross-sectional studies have reported pneumococcal carriage rates over 80% for Indigenous children living in Australia's Northern Territory. Considerable pneumococcal serotype diversity in this population has contributed to swift serotype replacement following introduction of the seven-valent pneumococcal conjugate vaccine in 2001 with no significant impact on overall pneumococcal carriage [4]or otitis media [5].

Transmission of pneumococci is believed to be via inhalation of contaminated respiratory droplets [6]. The role of contaminated hands and fomites in pneumococcal transmission and the potential impact of hygiene interventions on pneumococcal disease are unclear. In a previous report, pneumococcus was cultured from the hands of 37% of Indigenous children in a remote community, compared to 4% children attending urban child care centres suggesting that direct and indirect transmission may be important in the high rates of pneumococcal carriage and OM in remote Indigenous communities [7]. Thus, the aim of the current study was to determine the survival time of pneumococci on hands and fomites to test our hypothesis that the high prevalence of pneumococcal hand contamination was primarily a result of frequent inoculation, rather than survival.

## Methods

Ethical approval for the study was granted by the Human Research Ethics Committee of the Northern Territory Department of Health and Community Services and Menzies School of Health Research.

### Survival on Hands

Three adult non-Indigenous female volunteers (23–35 years of age) participated. Baseline swabs were taken to determine nasopharyngeal carriage of *S. pneumoniae*. The hands, which were clear of visible lesions, were cleaned using surgical hand preparation procedures as outlined in the Royal Darwin Hospital Infection Control Standards 2001; volunteers' nails, hands and forearms were cleaned with skin disinfectant (chlorhexidine) for 3 minutes, rinsed, and dried with clean towels. The palmar surface of the non-dominant hand was immediately inoculated with *S. pneumoniae *strain ATCC 49619 (serotype 19F) suspended in Serum broth (SB, 10% horse serum in Brain Heart Infusion Broth, Oxoid) and Mueller-Hinton broth (MH, Oxoid) at two concentrations each (Table 1).

**Table 1:** Number of pneumococcal colonies recovered from hands of three volunteers (H1 to H3), by inoculum density*, time post-inoculation, and type of suspension medium.

	Serum broth 435 CFU*			**Serum broth 242 CFU**			**Mueller-Hinton 546 CFU**			**Mueller-Hinton 331 CFU**		
	
**TIME**	H1	H2	H3	H1	H2	H3	H1	H2	H3	H1	H2	H3
**3 min**	70	342	16	38	117	26	127	227	14	38	117	26

**1 h**	7	7	6	1	8	1	0	0	0	0	0	0

**2 h**	0	3	1	1	2	0	0	0	0	0	0	0

**3 h**	5	7	1	0	3	5	0	0	0	0	0	0

Each of the SB dilutions (435 colony forming units (CFU) and 242 CFU), and MH dilutions (546 CFU and 331 CFU) were pipetted onto the skin of the distal, middle, and proximal phalanges and the carpophalangeal joints of digits 2 to 5 respectively (16 swab sites per volunteer). A cotton swab moistened in sterile saline was used to sample one site per finger at 3 minutes, 1 hour, 2 hours and 3 hours after initial inoculation; the moistened swab was rolled over the site for five seconds. The swabs were streaked directly onto horse blood agar plates (Oxoid). Between swabbing intervals, the volunteers remained in the laboratory; their hands did not make contact with any object and were not exposed to sunlight. After the final swabbing, the volunteers' hands were decontaminated in a biohazard cabinet according to surgical theatre hand preparation procedures from the Royal Darwin Hospital, Australia. After cleansing, hands were pressed onto sterile blood agar plates to confirm decontamination.

### Survival on glass and plastic

**Figure 1:**
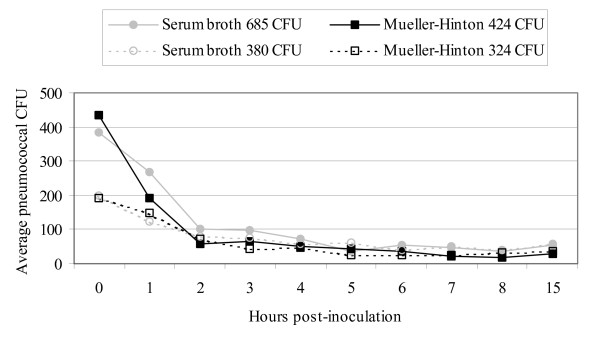
**Average pneumococcal colony forming units recovered from the glass plate at each hour post-inoculation, by inoculum density and type of suspension medium**. The original inoculum density is shown in legend.

The glass plate was sterilised by dry oven, and the plastic toy washed and sterilised with 70% ethanol prior to inoculation. They were placed on a grid template and inoculated in triplicate with two dilutions *S. pneumoniae *ATCC 49619 in SB and MH for each time point (Figure 1). The SB inocula used were 685 CFU and 380 CFU, and MH inocula were 424 CFU and 324 CFU. A cotton swab moistened in sterile saline was used to progressively sample grid sites on the glass plate (hourly) and the plastic toy (4-hourly); moistened swabs were rolled over the swab site for 5 seconds. Swabs were plated directly onto a blood agar plate and incubated overnight at 37°C in 5% CO_2_. Time 0 swabs (Figure 1) were taken directly after inoculation and used as the baseline measure.

## Results

At three minutes post-inoculation, pneumococcal counts from the hands of all volunteers were substantially lower than the initial inoculum (Table 1). There was considerable inter-volunteer variation; the greatest variation being for the higher SB inoculum density with 4% recovery for volunteer 3 compared to 79% recovery for volunteer 2. At the 3-minute time-point, H2 was consistently higher than the other volunteers demonstrating that this was not likely to be a methodological error. Survival was dependent upon the type of suspension medium; at one hour post-inoculation, pneumococci could not be cultured from either MH inoculum density. In contrast, for SB, 1 to10 CFU were recovered at the majority of samplings up to three hours post-inoculation for both inoculum densities. Pneumococcus was not found in nasopharyngeal swabs collected from the volunteers before or after exposure. Hand cultures after decontamination were also negative.

Viability of pneumococcus on a glass plate was monitored for 15 hours as illustrated in Figure 1. The coefficient of variation for the triplicate measures was below 30% for the vast majority of samplings. At time zero, immediately after inoculation of the plate, there was an average 66% recovery of pneumococci from the initial inoculum; the average time zero measurement was accordingly used as the baseline measure for the glass plate. In the first hour, recovery dropped an average 120 CFU (34%) for SB and MH suspensions, followed by a further average decrease of 105 CFU (57%) in the second hour. Beyond this point, less than 100 CFU were recovered from each grid up to the final sampling (15 hours post-inoculation). Similarly, for the plastic toy, there was an average loss of 266 CFU (85%) after four hours and an average of 12 to 48 colonies were recovered at the final eight-hour sampling.

## Conclusion

Hands and fomites are implicated as vehicles for transmission of a number of species of pathogenic bacteria and viruses. As a result, hygiene practices are promoted in domestic and commercial settings, including child care centres, primary health care and food preparation areas. However, less is understood about the survival of the more fastidious organisms, such as pneumococcus, and the potential impact of hand and surface cleaning on its transmission. We previously detected pneumococci on the hands of 37% of school-age children from a remote Indigenous community [7]. Whether pneumococci survive long periods or are frequently inoculated onto hands in nasal secretions could be important in informing the type of behavioural changes likely to be most effective in reducing transmission. Frequent inoculation would indicate a need for frequent hand washing and or practices that reduce hand inoculation. Long duration of survival may indicate a need for more rigorous (but possibly less frequent) hand washing.

Numerous studies report survival of pathogens on skin and fomites, including a systematic review of survival of nosocomial pathogens on inanimate surfaces [8]. For example, *Klebsiella pneumoniae*, *Proteus vulgaris *and *Pseudomonas aeruginosa *inoculated onto skin lost viability between 2 h to 6 h on most subjects [9]. Furthermore, *K. pneumoniae *and *P. vulgaris *survived on surfaces at least 24 h, and *P. aeruginosa *lasted 8 to 24 h [9]. *Haemophilus influenzae *type b (Hib) in Brain Heart Infusion Broth was recovered from cotton gauze, paper tissue, wax paper, and plastic toy for at least 12 h, with some specimens still positive at 48 h. Survival of Hib in mucous was up to 18 h [10]. The effect of nasal mucous on survival of pneumococci compared to liquid culture media is not known.

In the current study the low recovery of pneumococci in MH broth on hands after 3 minutes and complete attrition within 1 hour of inoculation, coupled with the survival of only small numbers in the viscous serum broth, suggests that survival on hands is unlikely to be the reason for the high prevalence of hand contamination among Indigenous children in remote communities. The most likely explanation for this finding is frequent inoculation of hands with contaminated nasal secretions. It is still plausible that hand contamination plays a (brief) role in inoculating other children directly, and indirectly through contamination via fomites. In contrast, attrition of pneumococci on fomites was independent of the type of suspension medium used. Pneumococcal numbers dropped to less than 100 CFU over 2 hours, and were still recovered at 15 hours. We do not know whether the numbers of recoverable pneumococci are sufficiently high to present a risk for acquisition. However, our results suggest that regular hand washing and timely cleansing or removal of contaminated fomites may be important in controlling transmission of pneumococci via these routes.

## Competing interests

The authors declare that they have no competing interests.

## Authors' contributions

HSV participated in the design and coordination of the study, assisted with the laboratory work, and drafted the manuscript. FC and JB assisted with the laboratory work and reviewed the manuscript. PSM and AJL participated in the design and coordination of the study, and helped to draft the manuscript. All authors read and approved the final manuscript.
